# Thermosensitive Hydrogel with Programmable, Self‐Regulated HIF‐1α Stabilizer Release for Myocardial Infarction Treatment

**DOI:** 10.1002/advs.202408013

**Published:** 2024-09-23

**Authors:** Kaicheng Deng, Yuyan Hua, Ying Gao, Houwei Zheng, Yangzi Jiang, Yaping Wang, Changyou Gao, Tanchen Ren, Yang Zhu

**Affiliations:** ^1^ State Key Laboratory of Transvascular Implantation Devices Department of Cardiology of The Second Affiliated Hospital School of Medicine Zhejiang University Hangzhou 310009 China; ^2^ MOE Key Laboratory of Macromolecular Synthesis and Functionalization Department of Polymer Science and Engineering Zhejiang University Hangzhou 310027 China; ^3^ Heart Regeneration and Repair Key Laboratory of Zhejiang Province Hangzhou 310009 China; ^4^ School of Biomedical Sciences Institute for Tissue Engineering and Regenerative Medicine The Chinese University of Hong Kong Hong Kong Special Administrative Region of China Hong Kong 999077 China; ^5^ Key Laboratory for Regenerative Medicine Ministry of Education The Chinese University of Hong Kong Hong Kong Special Administrative Region of China Hong Kong 999077 China; ^6^ Binjiang Institute of Zhejiang University Hangzhou 310053 China

**Keywords:** drug delivery, hydrogel, hypoxia‐inducible factor‐1α, myocardial infarction

## Abstract

HIF‐1α (hypoxia induced factor‐1α), a vital protective signal against hypoxia, has a short lifetime after myocardial infarction (MI). Increasing HIF‐1α stability by inhibiting its hydroxylation with prolyl hydroxylases inhibitors such as DPCA (1,4‐dihydrophenonthrolin‐4‐one‐3‐carboxylic acid) presents positive results. However, the optimal inhibitor administration profile for MI treatment is still unexplored. Here, injectable, thermosensitive hydrogels with programmable DPCA release are designed and synthesized. Hydrogel degradation and slow DPCA release are coupled to form a feedback loop by attaching pendant DPCA to polymer backbone, which serve as additional crosslinking points through π–π and hydrophobic interactions. Pendant carboxyl groups are added to the copolymer to accelerate DPCA release. Burst release in the acute phase for myocardial protection and extended near zero‐order release across the inflammatory and fibrotic phases with different rates are achieved. All DPCA‐releasing hydrogels upregulate HIF‐1α, decrease apoptosis, promote angiogenesis, and stimulate cardiomyocyte proliferation, leading to preserved cardiac function and ventricular geometry. Faster hydrogel degradation induced by faster DPCA release results in a HIF‐1α expression eight times of healthy control and better therapeutic effect in MI treatment. This research demonstrates the value of precise regulation of HIF‐1α expression in treating MI and other relevant diseases and provides an implantable device‐based modulation strategy.

## Graphical abstract

This work presents an injectable hydrogel using drug itself as crosslinking points, achieving programable release of HIF‐1α stabilizer for treating myocardial infarction (MI), and with which reports the desirable profile of myocardial HIF‐1α expression post MI.

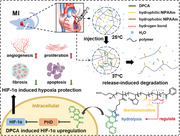

## 1 Introduction

Myocardial infarction (MI), as the predominant form of ischemic cardiovascular diseases, is a major threat to human health.^[^ ^1^, ^2^ ^]^ After MI, the sudden cessation of blood and oxygen supply induces metabolic variation and myocardial apoptosis, which further activates inflammatory response and fibrosis of the myocardium, increases the probability of heart failure.^[^ ^3^, ^4^ ^]^ Blocking the hypoxia‐induced adverse reactions has shown potential in restoring cardiac functions after MI. Myocardial protection under hypoxia is based on the increasing the expression level of hypoxia induced factor‐1α (HIF‐1α).^[^ ^5^, ^6^ ^]^ High expression of HIF‐1α has been reported to ameliorate apoptosis,^[^ ^7^, ^8^ ^]^ dampen inflammation reactions,^[^ ^9^, ^10^ ^]^ suppress fibrosis,^[^ ^11^ ^]^ and promote tissue repair post infarction.^[^ ^12^, ^13^ ^]^ In hypoxia condition, HIF‐1α pathway promotes angiogenesis by increasing the levels of critical angiogenic growth factors, including vascular endothelial growth factor (VEGF), stromal derived factor 1 (SDF1), angiopoietin 2 (ANGPT2), and stem cell factor (SCF).^[^ ^14^ ^]^ Furthermore, HIF‐1α has been reported to stimulate cardiomyocyte proliferative potential, which provided a new direction in hypoxic protection and regeneration of infarcted myocardium.^[^ ^15^ ^]^

However, HIF‐1α protein only possesses a half‐life of ≈5 min in normoxia condition^[^ ^16^ ^]^ and the expression level of HIF‐1α in ischemic myocardium reach peak at 24 h after infarction and then rapidly decreases.^[^ ^17^ ^]^ The central region of HIF‐1α contains an oxygen‐dependent degradation (ODD) domain, which can be hydroxylated on proline 402 (Pro402) and proline 564 (Pro564) by prolyl hydroxylase domain (PHD) proteins.^[^ ^18^ ^]^ Hydroxylated HIF‐1α is then recognized by von Hippel–Lindau protein (pVHL), and degraded via the ubiquitin‐proteasome pathway.^[^ ^17^, ^19^ ^]^ Elevated HIF‐1α level was found in hypoxic environment due to inhibited hydroxylation of HIF‐1α,^[^ ^20^ ^]^ showing the possibility of stabilizing HIF‐1α expression in ischemic myocardium. A small molecule PHD inhibitor, 1,4‐dihydrophenonthrolin‐4‐one‐3‐carboxylic acid (DPCA) was shown effective for HIF‐1α stabilization both in vitro and in small animals.^[^ ^13^ ^]^

Small molecular weight and poor solubility of free DPCA contributed to its short half‐life in vivo, hence limited the extension of HIF‐1α stabilization and the investigation of desirable release profile of DPCA for MI treatment. Encapsulating drug into carriers by physical blending or covalent bonding can be effective in improving safety and efficacy.^[^ ^21^ ^]^ Wang et al. embedded DPCA‐loaded nanoparticles in a matrix metalloproteinase (MMP) sensitive hydrogel for intramyocardial DPCA delivery.^[^ ^22^ ^]^ In this system, the release of DPCA was dependent on relatively high MMP level. As high MMP expression is associated with post‐MI inflammation and fibrosis, the release of DPCA based on MMP response could potentially lead to a delayed upregulation of HIF‐1α expression in principle, and limit the flexibility in adjusting the release rate of DPCA. Messersmith et al. covalently attaching DPCA to Polyethylene glycol (PEG), and took advantage of the π–π interaction between DPCA groups to form shear‐thinning hydrogels, which obtained greater therapeutic effects compared to free drug counterparts in cartilage repair.^[^ ^23^, ^24^ ^]^ However, when utilized the shear‐thinning hydrogel for myocardial infarction repair, the cyclic stress generated by myocardial motion could decrease its viscosity, potentially resulting in its dispersion.

Our previous research demonstrated the potential of injectable thermosensitive hydrogels based on poly(N‐isopropyl acrylamide) (PNIPAAm) in the treatment of myocardial infarction and developed strategies for side‐chain linking of active groups and accelerating hydrogel degradation through autocatalysis.^[^ ^25^, ^26^ ^]^ Therefore, in this study, we propose grafting DPCA onto the side chains of the PNIPAAm‐based copolymer via ester bonds, controlling the release rate of DPCA by regulating the strength of the autocatalytic effect. Here, the gelation of PNIPAAm is dominant, with the π–π recognition of the DPCA side groups assisting in gel formation. When DPCA starts to be released due to the breaking of ester bonds connected to the polymer backbone, the reduction in π–π interaction increases the hydrophilicity of the hydrogel, aiding the diffusion of DPCA from the hydrogel into the tissue. This design theoretically supports the long‐term stable release of DPCA, thereby stabilizing HIF‐1α expression levels. This autocatalytic process can be earlier activated and amplified by introducing carboxylic side groups into the polymer backbone. The release of DPCA depends solely on the properties of the hydrogel itself, and the presence of PNIPAAm prevents the hydrogel from disintegrating too fast after the decrease in DPCA interactions, especially in the beating myocardium.

**Figure 1:**
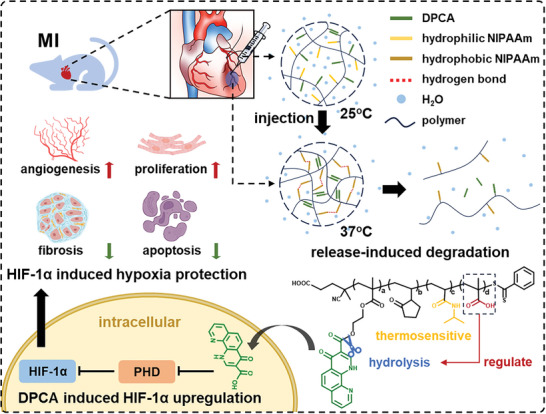
The design, function and application of the injectable release‐induced degradation thermosensitive hydrogel for HIF‐1α stabilization on myocardial infarction. Hydrogel formation is induced by hydrogen‐bonding interactions between hydrophobic NIPAAm at 37 °C and π–π interaction between DPCA. The pendant DPCAs on the polymer backbone enhanced gel formation by hydrophobic interaction. DPCA release can be initiated and controlled by the break of ester bonds. The released DPCA acts as an inhibitor of PHDs and maintains HIF‐1α stability to alleviate fibrosis and myocardial apoptosis, promote regeneration and angiogenesis.

Here, we prepared PNIPAAm‐based thermosensitive injectable hydrogels to program DPCA release. As shown in **Figure** **1**A, vinyl pyrrolidone (VP) was added to modulate lower critical solution temperature (LCST), DPCA was tethered on 2‐hydroxyethyl methacrylate (HEMA) units by esterification reaction, and methacrylic acid (MAA) was added for degradation acceleration and release control^[^ ^25^, ^26^ ^]^ (Figure 1A). We envision the molecular design can more flexibly control the release of DPCA, which provided a platform for exploring a desirable HIF‐1α expression profile in MI treatment.

## 2 Results

### 2.1 Synthesis and Characterization of DPCA‐Tethered Polymers

**Figure 2:**
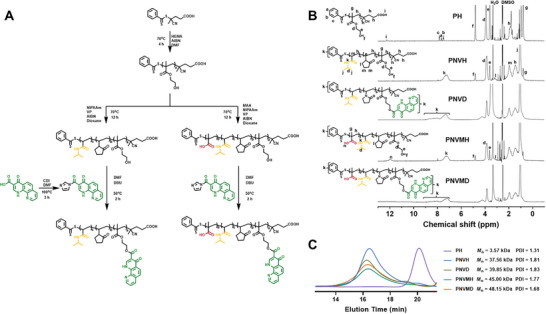
The synthesis and characterization of release‐induced degradation polymer. A) Synthesis route of PNVD and PNVMD. B) ^1^H NMR of polymers and their intermediates. C, SEC elution traces of polymers and their intermediates using HFIP as an eluent.

The polymer backbones of the DPCA‐tethered polymers P(NIPAAm‐*co*‐VP)‐*b*‐P(HEMA) (PNVH) and P(NIPAAm‐*co*‐VP‐*co*‐MAA)‐*b*‐P(HEMA) (PNVMH) were prepared by two‐step reversible addition‐fragmentation chain transfer (RAFT) polymerization as shown in **Figure** **2**A. DPCA was preactivated to switch the carboxyl group to imidazoline group, demonstrated with the disappearance of carboxyl hydrogen and the emergence of new peaks within benzene region in ^1^H NMR spectra (Figure S1, Supporting Information). The disappearance of the monomer double‐bond peaks and the emergence of their characteristic peaks in the ^1^H NMR spectrum (Figure 2B) confirmed the successful completion of polymerization and the removal of the unpolymerized monomers. The degree of polymerization (DP) of poly(HEMA) (PH) was calculated by determining the ratio of the hydrogen integral area on the hydroxyethyl (peak d and e) or hydroxyl group (peak f) of HEMA and that on the benzene (peak a, b, and c) of the chain transfer agent 4‐cyano‐4‐(phenylcarbonothioylthio)pentanoic acid (CPADB). Then the integral areas of the methine (peak d) of isopropyl on NIPAAm, the methylene next to N (peak l) on VP, and the carboxyl group (peak n) on MAA were utilized to calculate the DP of other monomers, using PH as the reference, respectively. As shown in **Table** **1**, the DPs of HEMA, NIPAAm, VP, and MAA were 30, 200, 30, and 30, respectively. The DPs were further verified by direct monitoring of the conversion rate of the monomer in the mixture after the reaction, thereby confirming the aforementioned conclusion. P(NIPAAm‐*co*‐VP)‐*b*‐P(HEMA‐DPCA) (PNVD) and P(NIPAAm‐*co*‐VP‐*co*‐MAA)‐*b*‐P(HEMA‐DPCA) (PNVMD) were synthesized by grafting DPCA onto the hydroxyl group of HEMA, which was demonstrated by the disappearance of the hydroxyl peak (peak f) on HEMA and the emergence of the distinctive peak of DPCA in the benzene region.

**Table 1:** Summary of *M*_n_, DP, and PDI of all synthesized polymers.

Polymer	DP						LC%	*M*_n_ ^b)^ [kDa]	*M*_n_ ^c)^ [kDa]	PDI ^c)^
	HEMA^a)^	NIPAAm^a)^	VP^a)^	MAA^a)^	DPCA^b)^	DPCA^c)^				
PH	30	‒	‒	‒	‒	‒	‒	4.18	3.57	1.31
PNVH	30	200	30	‒	‒	‒	‒	34.04	37.56	1.81
PNVD	30	200	30	‒	14.5	10.3	8.63	37.26	39.85	1.83
PNVMH	30	200	30	30	–	–	–	36.62	45.00	1.77
PNVMD	30	200	30	30	14	14.2	7.82	39.73	48.15	1.68

^a)^ Determined by ^1^H NMR;

^b)^ Determined by loading capacity (LC);

^c)^ Determined by SEC against PMMA standards.

The structural characterization of the polymers was further confirmed by Fourier Transform Infrared Spectroscopy (FTIR) and Ultraviolet–visible spectroscopy (UV–vis). As shown in Figure S2A,B (Supporting Information), the peaks observed near 3310, 1654, and 1544 cm^−1^ marked by orange box in PNVH, PNVD, and PNVMD can be attributed to the stretching vibration peaks of the N─H, C═O, and C─N bonds of the amide group in NIPAAm and VP, respectively. And the peaks observed near 3070 and 2970 cm^−1^ marked also by orange box can be attributed to the stretching vibration peaks of the C─H on the olefin and on the methyl group, respectively. These results indicated the successful synthesis of PNVH by RAFT extension of NIPAAm and VP using P(HEMA) as a macro‐CTA. The peaks observed near 1591 cm^−1^ marked by green box in PNVD and PNVMD can be attributed to the stretching vibration peak of the C═N double bond of the aromatic ring in DPCA, which indicated the successful tethering of DPCA. Compared with PNVD, the characteristic peaks of MAA in PNVMD were not obvious, but the peak at 3000–3500 cm^−1^ becoming broader and smoother indicated the presence of carboxyl groups. This result was further confirmed by the UV–vis spectroscopy as shown in Figure S2C (Supporting Information), where the characteristic absorption peak of both DPCA and PNVH appeared in that of PNVMD. The above results demonstrated the successful preparation of PNVD and PNVMD.

Molecular weights of the polymers were further detected by size exclusion chromatography (SEC). The number‐average molecular weight (*M*_n_) of PNVH and PNVD calculated by SEC was similar with *M*_n_ calculated by ^1^H NMR, but for PNVMH and PNVMD, *M*_n_ calculated by SEC was significantly larger than *M*_n_ calculated by ^1^H NMR which may be the result of the error caused by the enhanced intermolecular forces introduced by MAA. The increase of molecular weight after DPCA tethering also indicated the successful grafting of the pending group, and this increment can be used to estimate the amount of tethered DPCA.

### 2.2 Properties of the DPCA‐Loaded Thermosensitive Hydrogels

**Figure 3:**
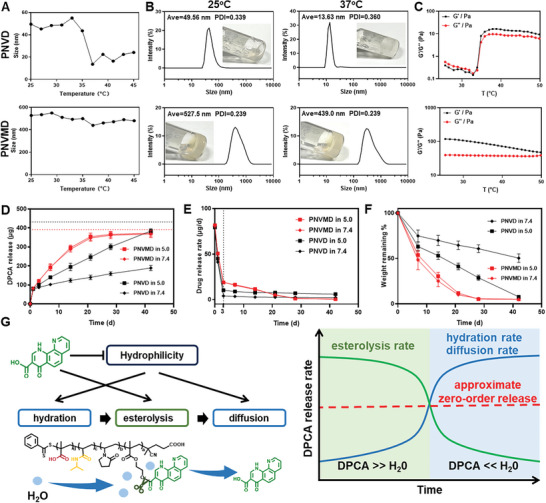
Properties of PNVD and PNVMD hydrogels. A) LCST of PNVD and PNVMD measured based on temperature‐induced particle size changes via DLS at a concentration of 1 mg mL^−1^ (LCST of PNVD was ≈35 °C, and no significant LCST in PNVMD). B) Particle size distribution of PNVD and PNVMD hydrogels (1 mg mL^−1^) at 25 and 37 °C. Inset: PNVD and PNVMD hydrogels (10 wt%) at 25 and 37 °C. C) The rheological properties of PNVD and PNVMD hydrogels at varying temperatures. D) Cumulative drug release of PNVD and PNVMD at pH 7.4 and 5.0. The dotted lines in red and black represent the theoretical drug loading of PNVMD and PNVD, respectively (*n* = 4). E) Drug release rate of PNVD and PNVMD at pH 7.4 and 5.0 (*n* = 4). F) Degradation of PNVD and PNVMD at pH 7.4 and 5.0 (*n* = 4). Data are presented as means ± SD. G) Schematic diagram showing the approximate zero‐order release of covalently bonded DPCA.

LCST of PNIPAAm is ≈32 °C, which can be adjusted by copolymerization with more hydrophilic or hydrophobic monomers.^[^ ^27^, ^28^ ^]^ LCST of thermosensitive polymers were measured by dynamic light scattering (DLS) with steady increased temperature, the inflection point of particle size indicated the sol–gel transition of the polymers (**Figure** **3**A; Figure S3, Supporting Information). Copolymerization of VP increased LCST to ≈35 °C while conjugation of DPCA lowered LCST. For PNVH and PNVMH, at T < 35 °C, the polymers are hydrophilic, presenting as free chain segments so the hydration diameter is hardly detected (Figure S3, Supporting Information). However, at 37 °C, NIPAAm transited into a hydrophobic state, thus instigating the formation of amphiphilic micelles with hydration diameters of 320.1 nm (PNVH) and 27.91 nm (PNVMH) at 37 °C as shown in Figure S3B (Supporting Information), respectively. For polymer solution with a concentration of 10 wt%, gelation at 37 °C could be clearly visualized by turbidity and fluidity change as shown in the insets of Figure S3B (Supporting Information). For PNVD, grafted DPCA groups presented as hydrophobic segments at room temperature, with a hydrated diameter of 49.56 nm. At 37 °C, hydrophobic interactions between the backbone and isopropyl segments induced shrinkage and curling of the polymer chain^[^ ^29^ ^]^ as evidenced by the reduction of the hydrated diameter to 13.63 nm and sol–gel transition of in10 wt% solution (Figure 3B). The hydration diameter of PNVMD was relatively stable with temperature, probably due to strong intermolecular interaction and confined polymer topology.^[^ ^29^ ^]^ LCST was also measured by rheological analysis, which showed the similar results. As shown in Figure 3C, and *G*′ and *G*″ of PNVD exhibited a notable increase at ≈35 °C, and *G*′ surpasses *G*″, indicating that the solution underwent a sol–gel transition. In contrast, *G*′ and *G*″ of PNVMD exhibited minimal variations with temperature, and *G*′ was greater than *G*″ in the entire temperature range tested, which indicated that the PNVMD kept its gel state. Furthermore, zeta potential and differential scanning calorimetry (DSC) analysis were conducted to evaluate the thermal transition of PNVD and PNVMD, and the results were consistent with observations above (Figure S4, Supporting Information). PNVD and PNVMD hydrogels exhibited desirable injectability, as they presented and maintained stable gel form after being injected into 37 °C water with regular syringes (Figure S5 and Movie S1, Supporting Information).

As an inhibitor of PHDs, DPCA stabilizes HIF‐1α and thereby modulates downstream pathways. The release profile of DPCA plays key roles in long‐term HIF‐1α expression in ischemic environment. As shown in Figure 3D–F, each group of hydrogels exhibited a significant burst release in the first 3 d due to the release of physically bonded DPCA. After that, the covalently bonded DPCA exhibited a gradual and sustained release with polymer degradation, which was tuned by the copolymer components (Figure 3D–F). The release profile was coped with the request for HIF‐1α stabilization in short‐term cardiomyocyte apoptosis and long‐term myocardial fibrosis after MI. A significant disparity in the rate of polymer weight loss and DPCA release was caused by the accelerated esterolysis with the introduction of MAA. As a result, the general release rate of PNVMD was significantly higher than PNVD. After 21 d, almost all DPCA in PNVMD was released in the presence of Lipase B, while less than 50% DPCA was released from PNVD. Drug release at pH 5.0 was further performed to elaborate on the divergence of release rates, the drug release of PNVD was accelerated at pH 5.0 while that of PNVMD did not change, demonstrating that the introduction of MAA induced weak acidity and consequently accelerated the hydrolysis of ester bonds via autocatalysis.

Interestingly, it can be observed in Figure 3D,E that the release rate of DPCA from PNVD after 3 d and that from PNVMD during days 3–14 were nearly constant, exhibiting approximate zero‐order release. This phenomenon was postulated to be due to the ingenious structure of the polymer. Briefly, the release of covalently bonded DPCA can be divided into three stages: hydration, esterolysis, and diffusion (Figure 3G). The rate of esterolysis was positively correlated to the amount of tethered DPCA, as the probability of esterolysis is higher. The rates of hydration and diffusion were negatively related to DPCA tethering amount as it affected the hydrophilicity of the polymer chain. Thus, at the initial stage, DPCA release was dominated by the faster rate of esterolysis but a slower rate of hydration and diffusion. At later stage, the concentration‐dominated esterolysis rate declined, yet the enhanced hydrophilicity increased the hydration and diffusion rate, resulting in an overall compensatory effect. This ultimately resulted in approximate zero‐order release. In addition, the DPCA loading capacity measured by drug release experiment was used to calculate the DPCA tethering rate backward, as shown in Table 1.

### 2.3 Cytocompatibility and In Vivo Degradability of the Hydrogels

Before intramyocardial injection, cytocompatibility and degradability of the hydrogels were evaluated. Cytocompatibility was studied by culturing H9C2 and L929 cells with extract medium of different hydrogels. No significant difference in cell viability were found among different groups (Figure S6, Supporting Information), indicating a negligible cytotoxicity of the resulted hydrogels, and a relatively low sensitivity of cardiomyocytes to the free drug. Myocardial injection in healthy rats showed that the hydrogels kept its original shape, did not induce severe inflammatory reaction or toxicity (Figure S7 and Table S1, Supporting Information) demonstrating the safety of long‐term DPCA release from hydrogel.

In vivo degradation experiments were performed by subcutaneous implantation as shown in Figure S8 (Supporting Information). At day 7, hydrogels were observed in PNVD and PNVMD groups from the subcutaneous tissue and HE staining images, while no hydrogels were observed in the other two groups, indicating the tethered DPCA groups increased the retention of PNVD and PNVMD hydrogels compared to the fast‐degrading PNVH hydrogel. At day 28, hydrogel was still observable in PNVD group, while PNVMD hydrogel disappeared, which is consistent with the degradation in vitro.

### 2.4 Programmed DPCA Release of Hydrogels for Cardiac Function Improvement

**Figure 4:**
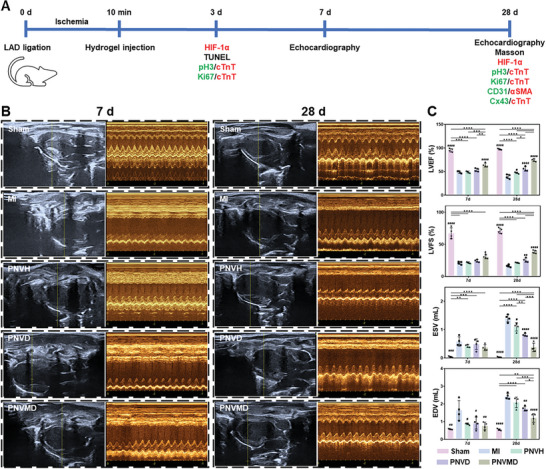
Evaluation of the cardiac functions after various treatments at different time points. A) Timeline of the animal study. Sham group (underwent thoracotomy, without LAD ligation); MI group (LAD ligation with saline injection); PNVH group (LAD ligation with PNVH hydrogel injection); PNVD group (LAD ligation with PNVD hydrogel injection); PNVMD group (LAD ligation with PNVMD hydrogel injection). B) The representative echocardiographic images for various groups at 7 d and 28 d, respectively. C) The statistical values of LVEF, LVFS, ESV, and EDV based on (B). Statistical significance was calculated using one‐way ANOVA with Tukey's posttest and data are presented as means ± SD. ^#^ *p* < 0.05, ^##^ *p* < 0.01, ^###^ *p* < 0.001, ^####^ *p* < 0.0001 versus MI group, ^∗^ *p* < 0.05, ^∗∗^ *p* < 0.01, ^∗∗∗^ *p* < 0.001, ^∗∗∗∗^ *p* < 0.0001 between the selected groups, respectively.

Based on the above results, hydrogels with good biocompatibility, injectability, and programmed DPCA release were applied on MI treatment. PNVH was introduced as a drug‐free control group, PNVMH was not included due to the similar component and property compared to PNVH. DPCA is highly hydrophobic hence not suitable for use alone, therefore a Free DPCA group was not included. As shown in the schedule of animal experiment (**Figure** **4**A), cardiac functions were measured by echocardiography at 7 and 28 d after hydrogel therapy (Figure 4B,D), with the statistical data of left ventricular ejection fraction (LVEF), left ventricular fractional shortening (LVFS), left ventricular end‐diastolic volume (EDV), and left ventricular end‐systolic volume (ESV) shown in Figure 4B. At Day 7, LVEF which is the key metric in cardiac function assessment, significantly decreased in MI group (46.65%) compared to Sham (94.98%), indicating cardiac dysfunction after myocardial infarction. At this time, PNVMD group significantly increased LVEF (63.66%), but PNVH and PNVD groups did not demonstrate a significant ability to improve cardiac function, due to their limited mechanical effects and drug release. After 28 d, further deterioration occurred in the infarcted myocardium without hydrogel intervention (MI group at Day 28), with LVEF falling to 39.53%. Hydrogel treatment played a role in improving cardiac function as reflected in LVEF. PNVH group showed a slightly higher LVEF (47.41%) than the MI group. PNVD group has a better improvement in cardiac function in response to higher LVEF (55.48%). PNVMD group significantly increased LVEF (73.99%) compared to PNVD group, demonstrating the faster release of DPCA can further improve heart function.

### 2.5 Effect of Hydrogels on HIF‐1α‐Induced Apoptosis and Regeneration of Cardiomyocytes

**Figure 5:**
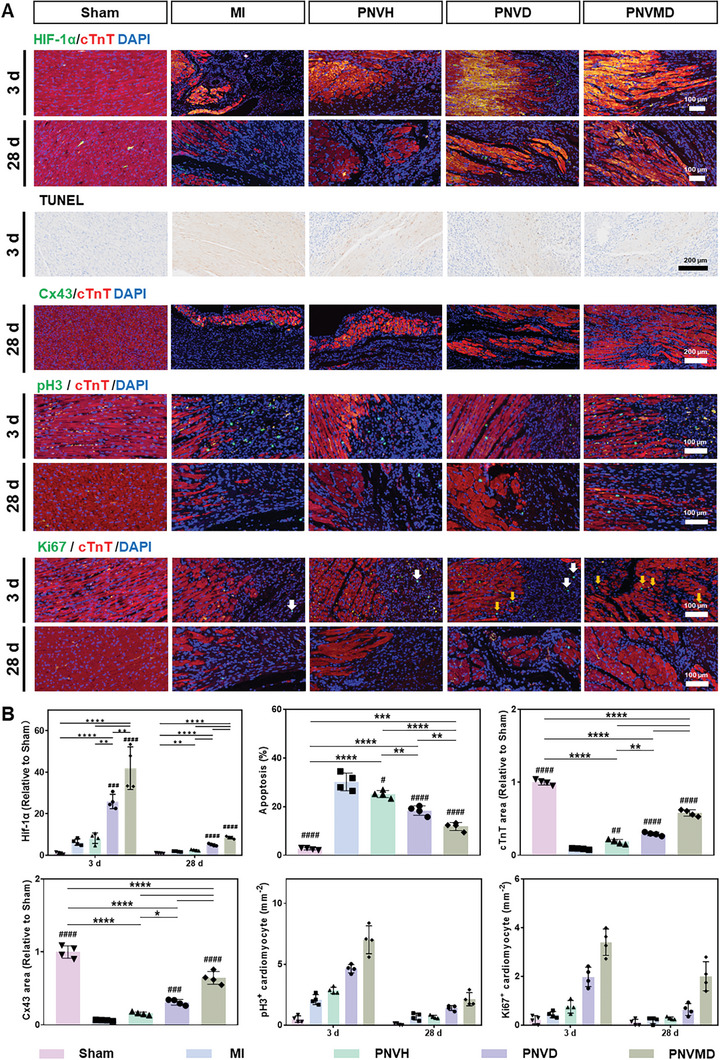
Effect of hydrogels on apoptosis and regeneration of cardiomyocytes. A) Histological evaluation of hearts sections after 3 d and 28 d of hydrogel treatment: representative immunofluorescence staining images of HIF‐1α (green), cTnT (red), and nuclei (blue) (3 d and 28 d); representative TUNEL staining images (only 3 d); representative immunofluorescence staining images of Cx43 (green), cTnT (red), and nuclei (blue) (only 28 d); representative immunofluorescence staining images of pH3 (green), cTnT (red), and nuclei (blue) (3 d and 28 d); representative immunofluorescence staining images of Ki67 (green), cTnT (red), and nuclei (blue) (3 d and 28 d). The white and yellow arrows represent positive markers in the fibrocytes and cardiomyocytes, respectively. B) Quantitative analysis of HIF‐1α expression, apoptosis, cTnT expression, Cx43 expression, pH3 expression and Ki67 expression based on corresponding immunofluorescence staining images and TUNEL staining images in (A). Statistical significance was calculated using one‐way ANOVA with Tukey's posttest and data are presented as means ± SD. ^#^ *p* < 0.05, ^##^ *p* < 0.01, ^###^ *p* < 0.001, ^####^ *p* < 0.0001 versus MI group, ^∗^ *p* < 0.05, ^∗∗^ *p* < 0.01, ^∗∗∗^ *p* < 0.001, ^∗∗∗∗^ *p* < 0.0001 between the selected groups, respectively.

As shown in **Figure** **5**, myocardial tissues showed minimal HIF‐1α expression in normal condition due to PHDs induced hydroxylation of HIF‐1α and following ubiquitination under normoxia microenviroment. At 3 d after MI, the HIF‐1α expression showed an increase owing to the hypoxic microenvironment caused by MI and the expression of HIF‐1α in drug‐free hydrogel (PNVH) showed no significant difference compared to MI group. In PNVD and PNVMD groups, HIF‐1α expression was significantly higher: ≈4.10 and 6.65 times higher in the PNVD and PNVMD groups compared to MI group; 25.86 and 42.96 times higher compared to sham group. As previously reported, HIF‐1α peaked at ≈24 h and then decreased^[^ ^17^ ^]^ in diseased hearts. The release of PHD‐inhibiting DPCA from PNVD and PNVMD were responsible for HIF‐1α stabilization. Higher HIF‐1α expression was observed in the PNVMD group compared to PNVD group, due to more DPCA was released in the beginning 3 d, which indicate proper acceleration of DPCA release is beneficial for early stabilization of HIF‐1α after MI. Then HIF‐1α expression at 28 d was also evaluated. Although HIF‐1α positive cells significantly decreased compared to day 3, the variations between groups showed the same trend that HIF‐1α expression in PNVD and PNVMD groups was significantly higher compared to MI (≈2.74 and 4.72 times higher in the PNVD and PNVMD groups compared to MI group), whereas there was no significant change in PNVH. The above results demonstrated that appropriate DPCA release upregulates HIF‐1α expression.

As shown in Figure 5, the ischemic injury in MI group caused significant cell death (30.18%) leading to a dramatic increase in cell density in the infarcted area compared to Sham group (2.64%). The percentage of apoptotic cardiac cells in PNVH slightly decreased (25.16%) compared to MI group. In PNVD and PNVMD groups, DPCA‐induced upregulation of HIF‐1α expression reduced apoptosis to 18.37% in PNVD group and 11.84% in PNVMD group. The faster release of DPCA in PNVMD group demonstrated a better role in anti‐apoptosis, which is consistent with the cardiac function results.

Expression of gap junction protein Cx43 and cardiac troponin T (cTnT), a key regulator of myocardial muscle contraction in ventricular cardiomyocytes were detected, as shown in Figure 5. Immunofluorescence staining 28 d post‐MI showed a significantly lower Cx43 density and tissue level discontinuity in cTnT expression, indicating a severe damage in infarcted myocardium from MI group. Hydrogel therapy increased the expression of Cx43 and cTnT, with the PNVMD group showing the most positive effect, indicating a healthier myocardium supported by hydrogel.

DPCA‐induced upregulation of HIF‐1α expression has shown promising results in regeneration,^[^ ^13^ ^]^ we thus studied its effects on cell proliferation in infarcted myocardium (Figure 5). Phospho‐Histone H3 (pH3) (the marker of G2 and M phases of the cell cycle) and Ki67 (the characterizers of the G1, S, G2, M phases), which can reflect cell proliferation were analyzed by immunofluorescent staining. The border zones and infarct zones of ischemic heart were observed, the signals of pH3 and Ki67 on cTnT positive cells that overlap with the nucleus were counted and shown in Figure 5, which demonstrated a similar trend as HIF‐1α expression. In normal rat hearts (Sham group), pH3 and Ki67 were scarcely expressed (0.49 pH3^+^ and 0.22 Ki67^+^ cardiomyocyte mm^−2^) and minimally expressed in MI (2.10 pH3^+^ and 0.43 Ki67^+^ cardiomyocyte mm^−2^) and PNVH (2.85 pH3^+^ and 0.75 Ki67^+^ cardiomyocyte mm^−2^) groups, due to the intrinsic poor multiplication capacity of mature cardiomyocytes. In PNVD and PNVMD groups, Ki67 and pH3 showed clear expression (4.63 pH3^+^ and 1.98 Ki67^+^ cardiomyocytes mm^−2^ in PNVD group, 7.01 pH3^+^ and 3.41 Ki67^+^ cardiomyocytes mm^−2^ in PNVDN group) with a trend similar with HIF‐1α expression. Then pH3 and Ki67 expression at 28 d was also evaluated. Although pH3^+^ and Ki67^+^ cardiomyocytes significantly decreased compared to day 3, the variations between groups showed the same trend that pH3 and Ki67 expression in PNVMD groups (2.12 pH3^+^ and 2.01 Ki67^+^ cardiomyocytes mm^−2^) as well as pH3 expression in PNVD group (1.36 pH3^+^ cardiomyocytes mm^−2^) were significantly increased compared to MI (0.64 pH3^+^ and 0.20 Ki67^+^ cardiomyocytes mm^−2^). The above results demonstrated that DPCA‐upregulated HIF‐1α expression can promote the expression of pH3 and Ki67, consistent with previous work that DPCA can promote regeneration of ear tissue by upregulating the expression of HIF‐1α.^[^ ^13^, ^30^ ^]^

### 2.6 Programmed DPCA Release of Hydrogels for MI Treatment by Reducing Fibrosis and Vascularization

**Figure 6:**
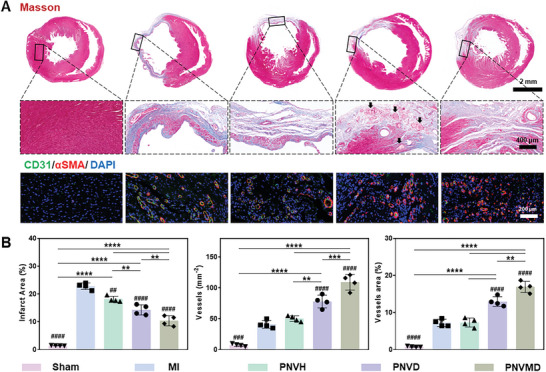
Myocardial infarction treatment by hydrogels 28 d after MI. A) Histological evaluation of therapeutic outcome by hydrogels: representative Masson's trichrome staining and representative immunofluorescence staining images of CD31 (green), αSMA (red), and nuclei (blue) of hearts sections. B) Quantitative analysis of infarct area and vascularization based on Masson's trichrome staining and corresponding immunofluorescence staining images in (A). Statistical significance was calculated using one‐way ANOVA with Tukey's posttest and data are presented as means ± SD. ^##^ *p* < 0.01, ^###^ *p* < 0.001, ^####^ *p* < 0.0001 versus MI group, ^∗^ *p* < 0.05, ^∗∗^ *p* < 0.01, ^∗∗∗∗^ *p* < 0.0001 between the selected groups, respectively.

Myocardial fibrosis usually happens at the late post‐MI stage.^[^ ^31^ ^]^ We detected fibrosis at 28 d post‐MI using Masson's trichrome staining^[^ ^32^ ^]^ shown in **Figure** **6**. Compared to MI group (22.83%), PNVH group slightly mitigated cardiac fibrosis (18.12%). DPCA intervention significantly reduced fibrosis (14.34% in PNVD group, 10.39% in PNVMD group). PNVMD group had the lowest fibrosis level due to appropriate drug release rate. Another 2 planes between the papillary muscle plane to the apex from the same rats showed the consistent trend (Figure S9, Supporting Information). This result was consistent with cardiac function results, confirming the treatment effect of desirable DPCA release.

Further magnified the infarcted zone with Masson's trichrome staining, we observed more pink lumps inside the heart tissue of PNVD group than PNVMD group, no such pink lump was visualized in other groups. The pink lumps are considered as the undegraded hydrogels.^[^ ^22^ ^]^ The results indicated that DPCA loading decelerated hydrogel degradation, with PNVD showed slower degradation than PNVMD hydrogel, which is consistent with the degradation in vitro and subcutaneous implantation.

Considering the contribution of HIF‐1α to vascularization,^[^ ^14^ ^]^ CD31 and α‐SMA were stained to evaluate vascularization of each group as shown in Figure 6. In the Sham group, minimal neovessels were observed, while a number of vessels were present in the MI and PNVH groups. This may be due to myocardial‐initiated self‐defense mechanisms to counteract the ischemic‐hypoxic microenvironment at the infarct site by inducing vascular regeneration.^[^ ^33^, ^34^ ^]^ The highest regenerated vessel density and number of vessels were observed in the PNVMD group, indicating that the synergistic effect of mechanical support and DPCA promotes vascular regeneration. These newly formed blood vessels hold immense importance in enhancing the infarct site during the later phases of the cardiac cycle.

## 3 Discussion

HIF‐1α plays a significant role in ischemic heart disease. In previous literatures, it was reported that HIF‐1α can provide a critical braking mechanism for post‐ischemic CF activation and proliferation through regulation of metabolism, ROS buildup, and intracellular signaling.^[^ ^20^ ^]^ It was also reported that cardiomyocyte‐specific overexpression of HIF‐1α ameliorates cardiomyocyte apoptosis via upregulation of the antioxidant gene Heme Oxygenase‐1 (HO‐1) and other downstream targets of HIF‐1α, including microRNAs, have also been reported to protect cardiomyocytes from apoptosis during ischemia.^[^ ^35^ ^]^ HIF‐1α is also reported to regulate key components involved in glycolysis, oxidative phosphorylation, and fatty acid metabolism, to rescue cardiomyocytes in hypoxic environments.^[^ ^9^, ^10^ ^]^ Furthermore, overexpression of HIF‐1α in cardiomyocyte upregulated critical angiogenic growth factors including VEGFA to promote collateral formation and improves cardiac function following myocardial infarction.^[^ ^36^, ^37^ ^]^ It has also been proven that overexpression of HIF‐1α can lead to regenerative wound healing in Swiss Webster mice after ear hole punch injury, which provide a new indication for future studies on regeneration by increasing expression of the HIF‐1α protein.^[^ ^13^ ^]^ Thus, it is potential to promote cardiomyocyte proliferation by stabilizing HIF‐1α.

Overexpression of HIF‐1α is generally achieved by intervening of PHDs inhibitors. The short‐term use of PHDs inhibitors is widely recognized to be beneficial for the heart. However, the long‐term effects of PHD inhibitor use on the ischemic heart still need to be explored.^[^ ^20^ ^]^ Although some reports suggested that sustained upregulation of HIF‐1α in transgenic mice can lead to heart failure,^[^ ^38^ ^]^ similar findings have not been observed in clinical trials, and long‐term inhibition of PHD alone does not worsen heart function.^[^ ^39^ ^]^ Additionally, in heart failure patients with specific comorbidities, long‐term use of PHDs inhibitors has significantly improved their cardiac function.^[^ ^40^, ^41^ ^]^ The mechanisms of HIF‐1α which involves multiple pathways are complex, and the specific mechanisms of its long‐term upregulation in the heart remain unclear, the results of this study provide some preliminary suggestions in this regard.

Tethering DPCA to the polymer effectively improves the drug loading and stability of the hydrogel, providing a long‐term, sustained, and stable release. Our strategy greatly increased the drug loading capacity and ensured a high concentration of the drug in the focal tissue for a long period of time, while in situ release also improved the efficiency of drug use and enhanced the biosafety of the material. The release rate can be regulated by the polymer components. DPCA molecules are used as the key switch of hydrogel formation to synergistically control the drug release and hydrogel state. The hydroxyl groups on HEMA in PNVH and PNVMH theoretically support bonding with different drug molecules with amino, carboxyl, and hydroxyl groups, to obtain corresponding prodrug hydrogels. Such strategy can be used to meet the needs of different diseases, providing a new insight for the design of programmable smart drug delivery system. The amphiphilic block copolymer structures of PNVD and PNVMD enable them to function as a prodrug while simultaneously physically encapsulating the drug, thereby facilitating two‐stage programmed releases at distinct rates to align with the disease's temporal requirements. In addition, the tunable release and degradation rate enabled the material to be more compatible with the repair pattern of the infarcted heart.^[^ ^42^ ^]^ Namely, the hydrogel provides mechanical support and continuously releases drug in the early stage of the disease and degrades in the late stage of the disease to avoid the adverse effects of prolonged hydrogel retention on recovery.^[^ ^43^, ^44^ ^]^ Furthermore, there is great potential in optimizing the properties of the presented hydrogel system to match different needs. The incorporation of diverse drug‐loading methodologies (e.g., host‐guest interactions) could support programmable releases for more temporal segments. Simply mixing PNVD and PNVMD with different drug release rates could also potentially facilitate the fabrication of multi‐segment drug release systems with distinct release profiles. Additionally, the drug release rate and amount are controllable by regulating the proportion of MAA and tethered drug in the copolymer, respectively.

Despite the promising results with the thermosensitive hydrogel‐based strategy for programmable, self‐regulated HIF‐1α stabilizer release, this study is still associated with following limitations. First, only a fast and a slow release DPCA‐tethered hydrogels were prepared, the optimal drug release profile could be further elucidated. Second, the monitoring time could be further extended to acquire the whole drug release situation of PNVD. Third, the effect of the hydrogel was only verified in a rat model, lacking validation in large animal experiments.

## 4 Conclusion

To explore the goal of stabilizing and regulating HIF‐1α expression after MI, we engineered PNVD and PNVMD hydrogels capable of programmable release of a HIF‐1α stabilizer. By modulating the interplay between ester bond hydrolysis of DPCA linked to the polymer backbone and the diffusion of free DPCA, we achieved rapid release during the acute phase and a prolonged, near zero‐order release throughout the inflammatory and fibrotic phases, with distinct release rates. The locally released DPCA preserved cardiac function and ventricular geometry by upregulating HIF‐1α, decreasing apoptosis, promoting angiogenesis, and stimulating cardiomyocyte proliferation. The release rate of DPCA from PNVMD is faster than from PNVD, resulting in higher levels of HIF‐1α in myocardial tissue, achieving levels up to eight times the healthy baseline, thereby resulting in superior therapeutic efficacy. In summary, this work highlights the optimization direction for HIF‐1α expression regulation in MI treatment and provides a hydrogel‐based regulation method.

## 5 Experimental Section

### Material

All chemicals were obtained from Sigma–Aldrich and used as received unless otherwise stated. VP and CPADB were purchased from Mackin. MAA was purchased from Aladdin. NIPAAm was recrystallized in n‐hexane and AIBN was recrystallized in methanol before use. Dulbecco'smodified Eagle's medium (DMEM) and fetal bovine serum (FBS) were purchased from Gibco, Cell counting kit‐8 (CCK‐8) was purchased from Beyotime H9C2 cell line (GNR 5) and L929 cell line (SCSP‐5039) were purchased from Cell Bank of Typical Culture Collection of Chinese Academy of Sciences.

### Synthesis of DPCA‐Im

DPCA was synthesized as previously described.^[^ ^45^ ^]^ A mixture of diethyl ethoxymethylenemalonate (2.32 mL, 11.4 mmol) and 8‐aminoquinoline (1.64 g, 11.4 mmol) was heated at 100 °C for 1 h. Then the mixture was cooled to 80 °C and recrystallized in 110 mL methanol, and the product was washed twice with 10 mL MeOH and dried under high vacuum to obtain diethyl[(quinolin‐8‐ylamino)methylidene]propanedioate (yield, 75%) as green‐brown needles. The purified product (1.10 g, 3.5 mmol) was added into 11 mL diphenylether, followed by heating at 250 °C and refluxing for 1 h. Then the mixture was cooled and filtrated to get the crude product, the crude product was further purified by washing with 2 mL cold Et_2_O and dried under high vacuum to obtain Ethyl 4‐oxo‐1,4‐dihydro‐1,10‐phenanthroline‐3‐carboxylate (yield, 46%) as a beige powder. The above product (1.00 g, 3.73 mmol) was added into 20 mL 10% (w/v) KOH, followed by heating at 110 °C and refluxing for 1 h. The mixture was cooled and residual diphenyl ether extracted using 14 mL petroleum ether. Then the crude product was further purified by precipitating in 20 mL 10% (w/v) HCl and washing with H_2_O. The purified DPCA was dried in vacuum (yield, 90%).

Then, above DPCA was activated by 1,1′‐carbonyldiimidazole for subsequent bonding to polymer via esterification. Typically, DPCA (1.0 g, 4.17 mmol) was added into 20 mL DMF, then 1,1′‐carbonyldiimidazole (2.0 g, 12.50 mmol) was added, and the mixture was stirred at 100 °C. After 3 h, the mixture was cooled to room temperature, then the product (DPCA‐Im) was separated and purified by washing with diethyl ether, dried in vacuum overnight (yield, 91%).

### Synthesis and Characterization of Thermal‐Sensitive Polymer

P(NIPAAm‐*co*‐VP)‐*b*‐P(HEMA) (PNVH) was prepared by two‐step reversible addition‐fragmentation chain transfer (RAFT) polymerization. First, P(HEMA) was synthesized by RAFT polymerization of HEMA in DMF using CPADB as a Chain transfer agent (CTA). Typically, HEMA (2.6 g, 20 mmol), AIBN (13.54 mg, 0.08 mmol), and CPADB (112 mg, 0.40 mmol) were dissolved in 20 mL DMF and then transferred to a Schlenk flask. After three freeze‐pump‐thaw cycles, the mixture was sealed and stirred at 70 °C. After 4 h, the mixture was quenched by exposure to the air. The mixture was diluted with THF, followed by precipitation in excess ice‐cold ethyl ether to get the crude product. The crude product was further purified by dissolving in DMF and precipitating in excess ice‐cold ethyl ether twice. The purified P(HEMA) was dried in vacuum (yield, 60%). Second, P(NIPAAm‐*co*‐VP)‐*b*‐P(HEMA) block copolymer was synthesized by RAFT extension of NIPAAm and VP using P(HEMA) as a macro‐CTA. Typically, NIPAAm (6.22 g, 55 mmol), VP (770 mg, 11 mmol), AIBN (3.3 mg, 0.02 mmol), and P(HEMA) (418 mg, 0.10 mmol) were dissolved in 33 mL Dioxane and then transferred to a Schlenk flask. After three freeze‐pump‐thaw cycles, the mixture was sealed and stirred at 70 °C. After 12 h, the mixture was quenched by exposure to the air. The mixture was diluted with THF, followed by precipitation in excess ice‐cold ethyl ether to get the crude product. The crude product was further purified by dissolving in DMF and precipitating in excess ice‐cold ethyl ether twice. The purified P(NIPAAm‐*co*‐VP)‐*b*‐P(HEMA) was dried in vacuum (yield, 41%).

P(NIPAAm‐*co*‐VP‐*co*‐MAA)‐*b*‐P(HEMA) was prepared by similar steps as P(NIPAAm‐*co*‐VP)‐*b*‐P(HEMA), the only difference was the addition of MAA during the second RAFT extension. Typically, NIPAAm (6.22 g, 55 mmol), VP (770 mg, 11 mmol), MAA (567.6 mg, 6.6 mmol), AIBN (3.3 mg, 0.02 mmol), and P(HEMA) (418 mg, 0.10 mmol) were dissolved in 36.3 mL dioxane and then transferred to a Schlenk flask. After three freeze‐pump‐thaw cycles, the mixture was sealed and stirred at 70 °C. After 12 h, the mixture was quenched by exposure to the air. The mixture was diluted with THF, followed by precipitation in excess ice‐cold ethyl ether to get the crude product. The crude product was further purified by dissolving in DMF and precipitating in excess ice‐cold ethyl ether twice. The purified P(NIPAAm‐*co*‐VP‐*co*‐MAA)‐*b*‐P(HEMA) was dried in vacuum (yield, 39%).

P(NIPAAm‐*co*‐VP)‐*b*‐P(HEMA‐DPCA) (PNVD) was prepared by the esterification of P(NIPAAm‐*co*‐VP)‐*b*‐P(HEMA) through the reaction between hydroxyl groups on the P(HEMA) block with an excess amount of DPCA‐Im. Typically, P(NIPAAm‐*co*‐VP)‐*b*‐P(HEMA) (340 mg, 0.01 mmol) was added in 15 mL DMF, and then the mixture was bubbled with nitrogen. After 30 min, DPCA‐Im (116 mg, 0.40 mmol) and DBU (60 mg, 0.40 mmol) were added, then the mixture was stirred under nitrogen atmosphere at 45 °C. After 3 h, the mixture was precipitated in diethyl ether (with 1% acetic acid) and dissolved in DMF, followed by purification using ultrafiltration centrifugation with a MWCO of 10 kDa. Then the purified product was precipitated in diethyl ether and dried in vacuum (yield, 89%).

P(NIPAAm‐*co*‐VP‐*co*‐MAA)‐*b*‐P(HEMA‐DPCA) (PNVMD) was prepared by similar steps as P(NIPAAm‐*co*‐VP)‐*b*‐P(HEMA‐DPCA). Briefly, P(NIPAAm‐*co*‐VP)‐*b*‐P(HEMA‐DPCA) (366 mg, 0.01 mmol) was added in 15 mL DMF, and then the mixture was bubbled with nitrogen. After 30 min, DPCA‐Im (116 mg, 0.40 mmol) and DBU (60 mg, 0.40 mmol) were added, then the mixture was stirred under nitrogen atmosphere at 45 °C. After 3 h, the mixture was precipitated in diethyl ether (with 1% acetic acid) and dissolved in DMF, followed by purification using ultrafiltration centrifugation with a MWCO of 10 kDa. Then the purified product was precipitated in diethyl ether and dried in vacuum (yield, 92%).

^1^H NMR spectra were recorded on a Avance III 400 m spectrometer (Bruker, Germany) using DMSO‐*d*6 as the solvents and TMS as the internal reference.

UV–vis spectroscopy was recorded on UV 2700i (Shimadzu, Janpan). Fourier Transform Infrared Spectroscopy (FTIR) was recorded on Nicolet 6700 (Themo Fisher, USA).

Molecular weights (*M*_n_) and polydispersity index (PDI) of polymers were determined by a size‐exclusion chromatography (SEC) equipment composed of Waters 1515 isocratic HPLC pump, Waters 2414 refractive index detector, Waters 2707 autosampler, and PL HFIPgel column (MW between 200 and 2000000). Hexafluoroisopropanol was employed as eluent with a flow rate of 0.5 mL min^−1^. Commercial poly(methylmethacrylate) (PMMA) was used as the calibration standards.

### Preparation and Characterization of Hydrogels

Polymers were dissolved in PBS (pH 7.4, 1X) at room temperature to prepare hydrogel (10 wt%). Hydrogels were heated to 37 °C to observe the sol–gel transition.

A polymer solution was prepared using PBS (pH 7.4, 1X) at a concentration of 1 mg mL^−1^. Dynamic light scattering (DLS) was used to determine the hydrodynamic diameter distribution, polydispersity, and zeta potential on a Malvern Nano ZS Zetasizer (Malvern, UK). Variation with temperature was measured from 25 to 45 °C and one point every 2 °C by DLS.

Rheology studies were conducted on a MARS 60 rheometer (HAAKE, Germany) to observe viscosity changes in the hydrogels during the temperature induced sol–gel transition. The polymer solutions (10 wt% in PBS) were placed between two parallel plates. With a temperature sweep from 25 to 45 °C and a heating rate of 2 °C min^−1^, the shear storage modulus *G*′ and the loss modulus *G*″ were collected as a function of temperature at a fixed strain of 1% and a frequency of 1 Hz.

### In Vitro Drug Release and Degradation of Hydrogels

In vitro drug release and degradation of hydrogels was detected in PBS at pH 7.4 and 5.0, respectively. Fifty microliters hydrogel (10 wt%) was added into the upper mini cup of Slide‐A‐Lyzer MINI Dialysis Cup (0.5 mL, 3.5 kDa MWCO, Thermo Fisher Scientific, USA), while 14 mL PBS containing with 0.02 wt% Candida antarctica Lipase B^[^ ^45^ ^]^ (Sigma–Aldrich, USA) was added into the lower conical tubes. Then, the MINI Dialysis Cups were kept in a horizontal laboratory shaker at 37 °C with an oscillation speed of 200 rpm. At predetermined time intervals, the solution in the lower conical tube of MINI Dialysis Cup was measured at 262 nm using a UV–vis spectrometer (UV‐2700i, Shimadzu, Japan), and the drug concentration of DPCA was calculated based on a standard curve. The residual hydrogel in the upper mini cup was weighed. The data of drug release and degradation at each point was summarized into a curve.

### Biocompatibility of Hydrogels

Male SD rats were injected intraperitoneally with 1% sodium pentobarbital for anesthesia, followed by endotracheal intubation and ventilation for assistance. The rats were placed on their backs for a left thoracotomy and pericardectomy to expose their hearts. Ten minutes after LAD ligation, 50 µL PNVMD hydrogel was injected into infarcted LV of rats, the rats in the Sham group were injected with 50 µL of saline as a control (*n* = 3). After injection, the chest cavity was sutured using 3‐0 silk sutures in the muscles and skin. After 14 d, the rats were euthanized and whole blood was taken for routine and blood biochemistry tests, followed by removal of the heart, liver, spleen, lungs, and kidneys for H&E staining to assess the biocompatibility of the hydrogel.

### In Vivo Degradation of Hydrogels

This animal experiments were approved by the Guidelines of Animal Care and Use Committees of Zhejiang Academy of Medical Sciences (ZJCLA‐IACUC‐20010499). Male Sprague–Dawley (SD) rats were anesthetized with 1% sodium pentobarbital by intraperitoneal injection. PNVH, PNVD, and PNVMD were preconfigured into 10 wt% solution in PBS. Fifty microliters PBS, PNVH PNVD, and PNVMD pregel solutions were injected subcutaneously at the back of rats (*n* = 4). At day 7 and 28, the rats were executed, and the dorsal skin was peeled off to observe the injection site of hydrogel. Then the skins were fixed with paraformaldehyde, embedded in paraffin, sectioned and H&E staining, and the histomorphology was observed to evaluate the degradation.

### In Vivo Hydrogel Injection Studies

These animal experiments were approved by the Guidelines of Animal Care and Use Committees of Zhejiang Academy of Medical Sciences (ZJCLA‐IACUC‐20010498). Male SD rats were injected intraperitoneally with 1% sodium pentobarbital for anesthesia, followed by endotracheal intubation and ventilation for assistance. The rats were placed on their backs for a left thoracotomy and pericardectomy to expose their hearts. The left anterior descending (LAD) coronary artery was then ligated using a 6‐0 silk suture, ≈2–3 mm from its origin between the left atrium and the pulmonary artery conus to induce infarction of the left ventricle. Successful establishment of MI model was shown through the luster loss and pale color shift of LV myocardium.

Rats were divided into five groups in random (*n* = 4): 1) Sham group (underwent thoracotomy, without LAD ligation); 2) MI group (LAD ligation with saline injection); 3) PNVH group (LAD ligation with PNVH injection); 4) PNVD group (LAD ligation with PNVD injection); and 5) PNVMD group (LAD ligation with PNVMD injection). Ten minutes after LAD ligation, 50 µL corresponding pregel solutions for each group or saline were injected into infarcted LV of rats. After injection, the chest cavity was sutured using 3‐0 silk sutures in the muscles and skin.

Cardiac functions at 7 d and 28 d post MI were assessed using echocardiography (VisualSonics, Canada), M‐mode echocardiographic and 2D images in a parasternal short and long axis were recorded. Left ventricular ejection fraction (LVEF), left ventricular fractional shortening (LVFS), left ventricular end‐diastolic volume (EDV), and left ventricular end‐systolic volume (ESV) were calculated as previously described.^[^ ^4^ ^]^

Rat hearts were harvested at 3 and 28 d post MI, fixed with 4% paraformaldehyde, embedded in paraffin, and sectioned by 4 µm. The heart sections were then dewaxed in xylene, hydrated in gradient ethanol, and washed in water. To get antigen retrieval, sections were soaked in 0.01 m sodium citrate buffer solution (pH 6.0) and boiled in pressure cooker for 15 min. After being blocked in 5%FBS for 30 min, the sections were incubated with primary antibodies overnight at 4 °C as follows: mouse HIF‐1α antibody (ThermoFisher, MA1‐16504, 1:50), mouse α‐SMA antibody (Boster, BM0002, 1:1000), mouse cardiac troponin T antibody (abcam, ab8295, 1 µg mL^−1^), rabbit connexin 43/GJA1 antibody (abcam, ab11370, 1:1000), rabbit CD31 antibody (abcam, ab182981, 1:2000), rabbit Ki67 antibody (abcam, ab16667, 1:200), rabbit Histone H3 (phospho S10) antibody (abcam, ab177218, 1:1000). The sections were washed with PBS three times, 5 min each, and then incubated with corresponding secondary antibodies for 1 h at room temperature as follows: goat anti‐rabbit IgG H&L (Alexa Fluor 488) (abcam, ab150077,1:500), goat anti‐rabbit IgG H&L (Alexa Fluor 594) (abcam, ab150080, 1:500), goat anti‐mouse IgG H&L (Alexa Fluor 488) (abcam, ab150113, 1:500), goat anti‐mouse IgG H&L (Alexa Fluor 594) (abcam, ab150116, 1:500). After being washed with PBS three times, the sections were stained with DAPI for 10 min.

Besides similar preliminary processing, TUNEL staining (Colorimetric TUNEL Apoptosis Assay Kit, beyotime, C1091) and Masson's trichrome staining (Masson's Trichrome Staining Kit, beyotime, C0189S) were performed according to the instructions.

Stained slides were observed using an Olympus IX51 microscope. Images were captured using DP2‐BSW software (Olympus, VS200, USA), and analyzed by ImageJ software.

### Statistics and Reproducibility

Statistical analysis was performed using Graphpad Prism 8. Results are shown as mean±standard deviation. One‐way analysis of variance (ANOVA) was used for multiple comparisons unless stated otherwise. *p* < 0.05 was considered statistically significant.

## Conflict of Interest

The authors declare no conflict of interest.

## Supporting information



Supporting Information

Supplemental Movie 1

## Data Availability

The data that support the findings of this study are available from the corresponding author upon reasonable request.
