# Supplementary material for: Thermosensitive Hydrogel with Programmable, Self‐Regulated HIF‐1α Stabilizer Release for Myocardial Infarction Treatment
Source: Adv Sci (Weinh). 2024 Sep 23;11(43):2408013. doi: 10.1002/advs.202408013 (PMC11578370; doi:10.1002/advs.202408013)
Supplement: Supplementary file 1 — Supporting Information [file ADVS-11-2408013-s001.pdf]

ADVANCED SCIENCE
Open Access

Supporting Information

for *Adv. Sci.*, DOI 10.1002/advs.202408013

Thermosensitive Hydrogel with Programmable, Self-Regulated HIF-1α Stabilizer Release for Myocardial Infarction Treatment

Kaicheng Deng, Yuyan Hua, Ying Gao, Houwei Zheng, Yangzi Jiang, Yaping Wang, Changyou Gao, Tanchen Ren* and Yang Zhu**

Supporting information

Thermosensitive hydrogel with programmable, self-regulated HIF-1α stabilizer release for myocardial infarction treatment

Kaicheng Deng, Yuyan Hua, Ying Gao, Houwei Zheng, Yangzi Jiang, Yaping Wang, Changyou Gao, Tanchen Ren, Yang Zhu

Contents

Figure S1. ^{1}H NMR of DPCA and DPCA-Im.

Figure S2. FTIR and ultraviolet-visible spectroscopy of polymers.

Figure S3. Properties of the PNVH and PNVMH hydrogels.

Figure S4. LCSTs of PNVD and PNVMD evaluated by zeta potential and DSC analysis.

Figure S5. The injectability of PNVD and PNVMD hydrogels.

Figure S6. Cytocompatibility of PNVH, PNVD and PNVDM hydrogels on H9C2 (left) and L929 (right).

Figure S7. Influence of PNVMD on rat haptorenal function and histology of important organs.

Figure S8. *In vivo* degradation in the subcutaneous tissue at day 7 and 28.

Figure S9. Representative Masson's trichrome staining images of left ventricle 28 d after MI.

Table S1. Blood routine examination of rats with or without PNVMD injection.

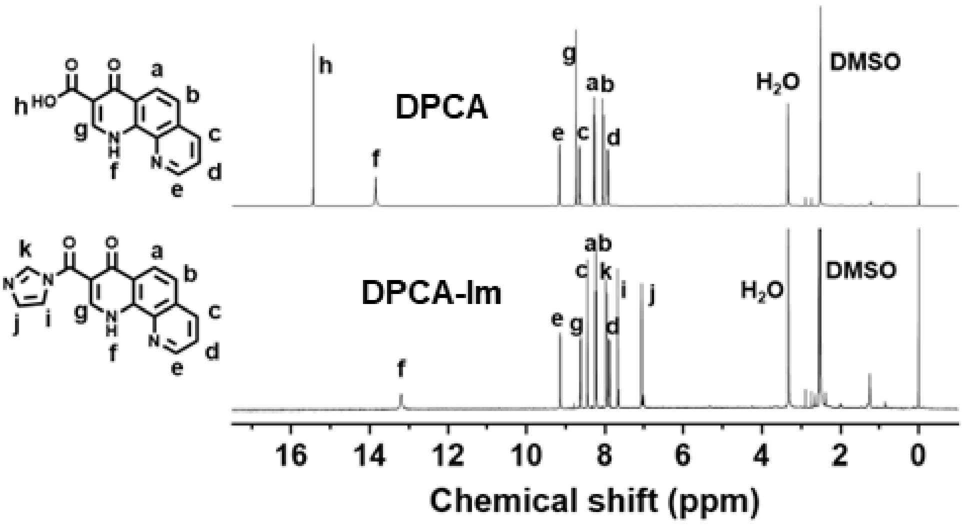

Figure S1. ^{1}H NMR of DPCA and DPCA-Im.

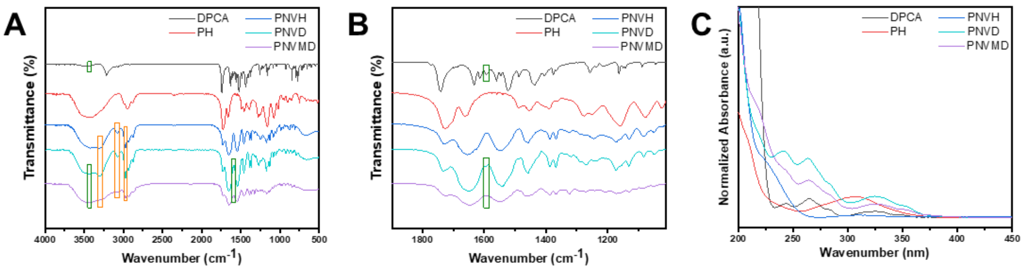

Figure S2. FTIR and ultraviolet-visible spectroscopy of polymers.

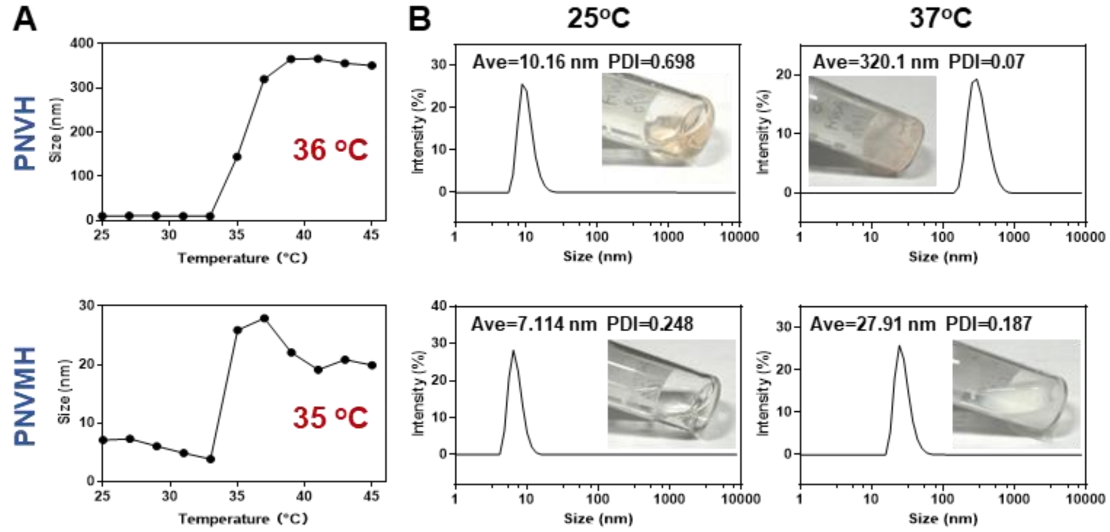

Figure S3. Properties of the PNVH and PNVMH hydrogels. A, LCST of PNVH and PNVMH measured based on temperature-induced particle size changes via DLS at a concentration of 1 mg/mL (LCST of PNVH and PNVMH was about 35ºC and 36ºC, respectively). **B,** Particle size distribution of PNVH and PNVMH hydrogels (1 mg/mL) at 25 and 37ºC. Inset: PNVH and PNVMH hydrogels (10 wt%) at 25 and 37ºC.

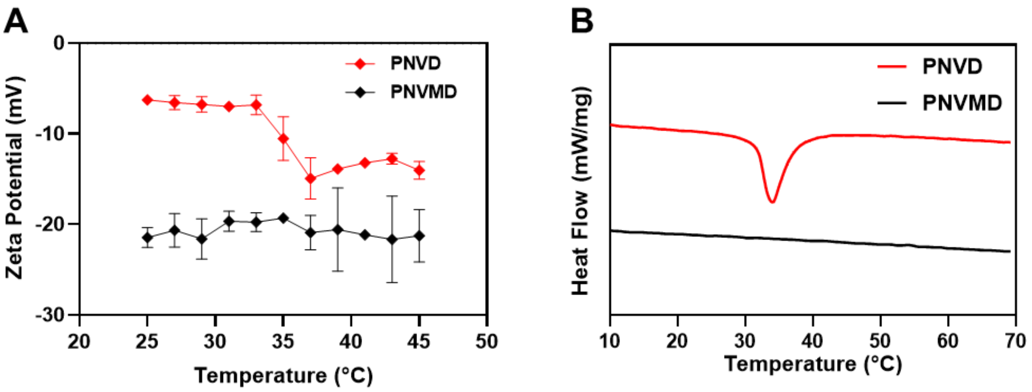

Figure S4. LCSTs of PNVD and PNVMD evaluated by (A) zeta potential and (B) DSC analysis.

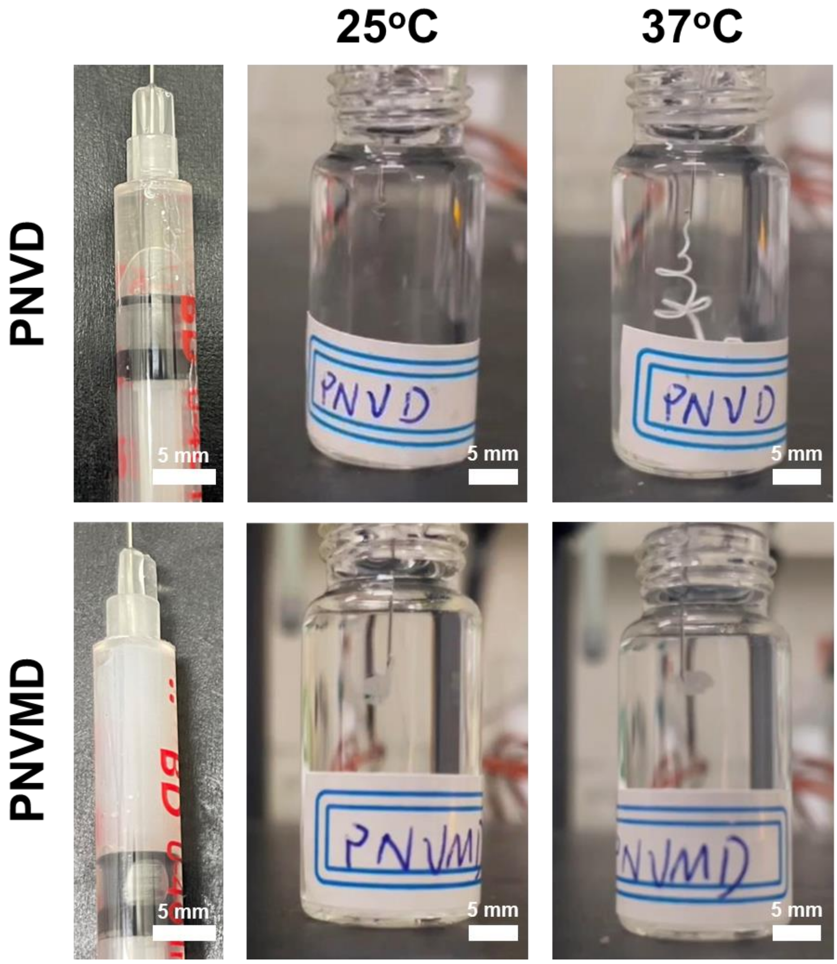

Figure S5. The injectability of PNVD and PNVMD hydrogels.

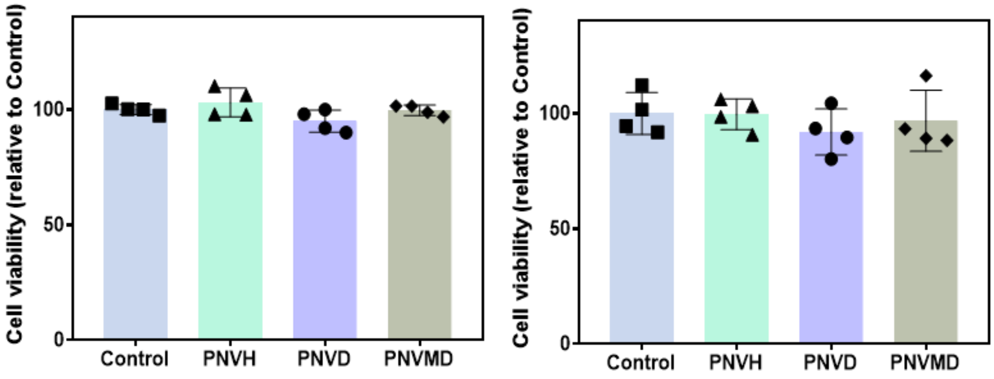

Figure S6. Cytocompatibility of PNVH, PNVD and PNVDM hydrogels on H9C2 (left) and L929 (right).

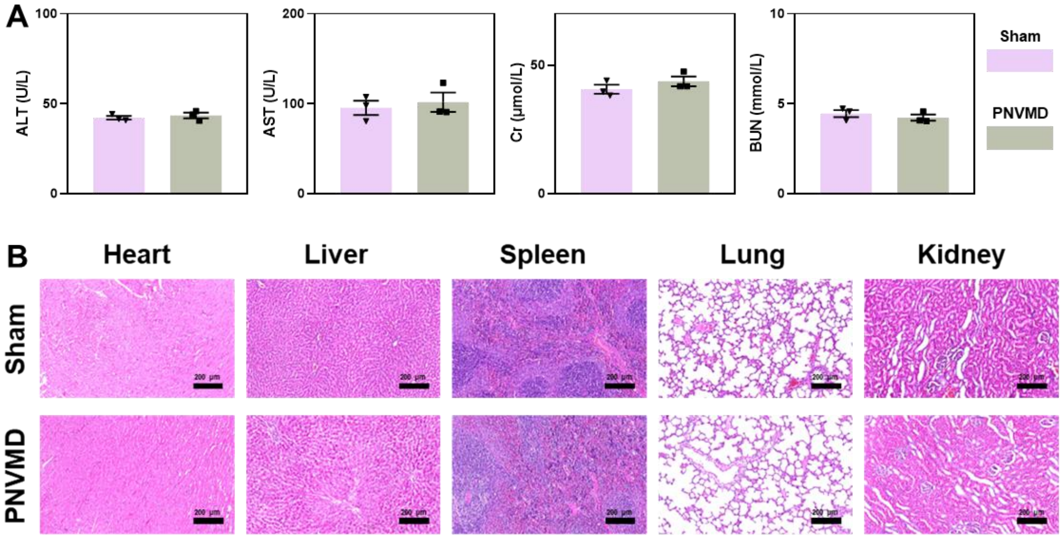

Figure S7. Influence of PNVMD on rat haptorenal function and histology of important organs. (A) Levels of hepatorenal function markers, n = 3. (B) Histopathologic examination of vital organs with H&E staining, n = 3.

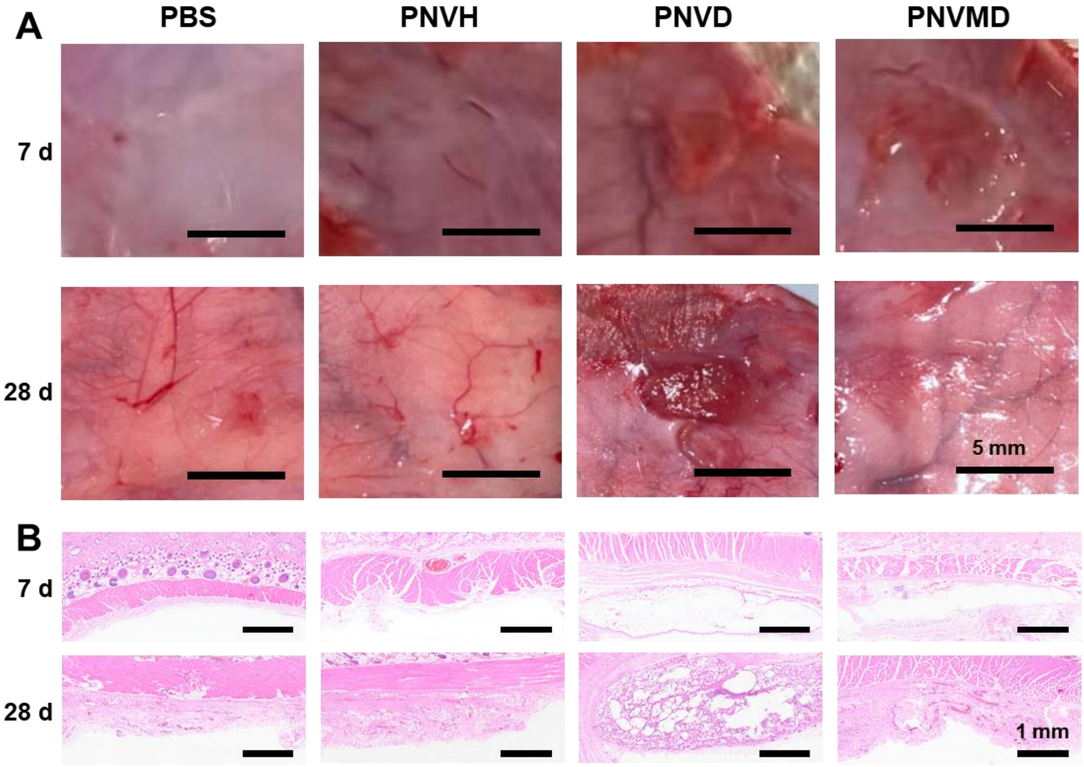

Figure S8. *In vivo* degradation in the subcutaneous tissue at day 7 and 28. **A,** the representative macro pictures illustrate the subcutaneous injection of hydrogels at day 7 and 28. **B,** Representative H&E staining of the tissue around the hydrogels at day 7 and 28.

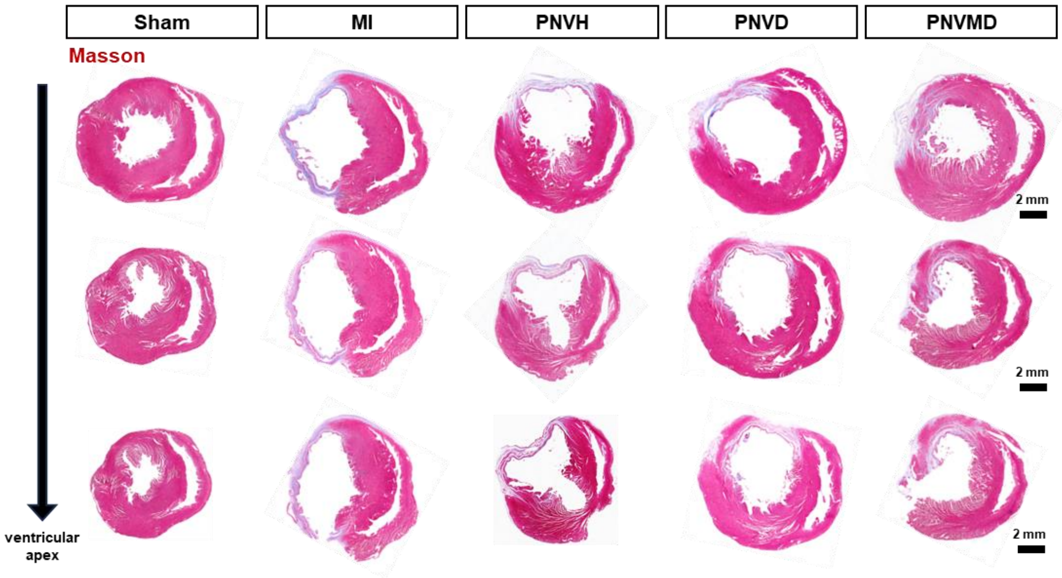

Figure S9. Representative Masson's trichrome staining images of left ventricle 28 d after MI.

Table S1. Blood routine examination of rats with or without PNVMD injection

Samples	WBC (10^9/L)	Neu # (10^9/L)	Lym # (10^9/L)	Mon # (10^9/L)	Eos # (10^9/L)	Bas # (10^9/L)	Neu % (%)	Lym (%)	Mon (%)	Eos (%)	Bas (%)	RBC (10^12/L)
Sham	8.74	1.16	6.01	0.39	1.18	0	13.4	68.7	4.5	13.4	0	4.92
	13.32	0.96	10.64	0.38	1.33	0.01	7.2	79.9	2.9	10	0	5.63
	11.13	1.91	7.49	0.45	1.28	0	17.2	67.3	4	11.5	0	4.83
PNVMD	14.89	2.92	8.66	0.78	2.51	0.02	19.6	58.2	5.2	16.9	0.1	4.86
	12.6	1.7	8.96	0.79	1.14	0.01	13.5	71.1	6.2	9.1	0.1	4.97
	14.56	3.55	8.56	0.73	1.69	0.03	24.4	58.8	5	11.6	0.2	5.02

Table S1. Blood routine examination of rats with or without PNVMD injection (Continued)

Samples	HGB (g/L)	HCT (%)	MCV (fL)	MCH (pg)	MCHC (g/L)	RDW-CV (%)	RDW-SD (fL)	PLT (10^9/L)	MPV (fL)	PDW (%)	PCT (%)
Sham	119	28.6	58.2	24.1	414	14.2	37.3	775	6.8	15.6	0.526
	131	31	55.1	23.3	423	13.3	33.3	861	6.2	15.2	0.532
	118	28.3	58.6	24.4	416	12.9	34	878	6.2	15.2	0.547
PNVMD	117	28.5	58.6	24	409	15.3	40.5	963	6.8	15.2	0.657
	115	27.9	56.1	23.1	412	14.7	37	992	7.6	15.7	0.751
	127	30.2	60.2	25.3	421	14.2	38.1	802	7.1	15.4	0.572
